# Multi‐level insights into the immuno‐oncology‐microbiome axis: From biotechnology to novel therapies

**DOI:** 10.1002/imt2.240

**Published:** 2024-09-07

**Authors:** Zheshun Pi, Weici Liu, Chenghu Song, Chuandong Zhu, Jiwei Liu, Lu Wang, Zhao He, Chengliang Yang, Lei Wu, Tianshuo Liu, Zijie Geng, Scott J. Tebbutt, Ningning Liu, Yuan Wan, Faming Zhang, Wenjun Mao

**Affiliations:** ^1^ Department of Thoracic Surgery The Affiliated Wuxi People's Hospital of Nanjing Medical University, Wuxi People's Hospital, Wuxi Medical Center, Nanjing Medical University Wuxi China; ^2^ Department of Microbiota Medicine Medical Center for Digestive Diseases, The Second Affiliated Hospital of Nanjing Medical University Nanjing China; ^3^ Center of Clinical Research, The Affiliated Wuxi People's Hospital of Nanjing Medical University, Wuxi People's Hospital, Wuxi Medical Center, Nanjing Medical University Wuxi China; ^4^ The Pq Laboratory of Biome Dx/Rx, Department of Biomedical Engineering Binghamton University Binghamton New York USA; ^5^ Department of Radiotherapy The Second Hospital of Nanjing, Nanjing University of Chinese Medicine Nanjing China; ^6^ State Key Laboratory of Systems Medicine for Cancer Center for Single‐Cell Omics, School of Public Health, Shanghai Jiao Tong University School of Medicine Shanghai China; ^7^ Centre for Heart Lung Innovation and PROOF Centre of Excellence, Providence Research, St Paul's Hospital Vancouver British Columbia Canada; ^8^ Division of Respiratory Medicine, Department of Medicine University of British Columbia Vancouver British Columbia Canada; ^9^ National Key Laboratory for Novel Software Technology China & School of Artificial Intelligence, Nanjing University Nanjing China; ^10^ School of Information Science and Technology, University of Science and Technology of China Hefei China

## Abstract

The multifaceted interactions among the immune system, cancer cells and microbial components have established a novel concept of the immuno‐oncology‐microbiome (IOM) axis. Microbiome sequencing technologies have played a pivotal role in not only analyzing how gut microbiota affect local and distant tumors, but also providing unprecedented insights into the intratumor host‐microbe interactions. Herein, we discuss the emerging trends of transiting from bulk‐level to single cell‐ and spatial‐level analyses. Moving forward with advances in biotechnology, microbial therapies, including microbiota‐based therapies and bioengineering‐inspired microbes, will add diversity to the current oncotherapy paradigm.

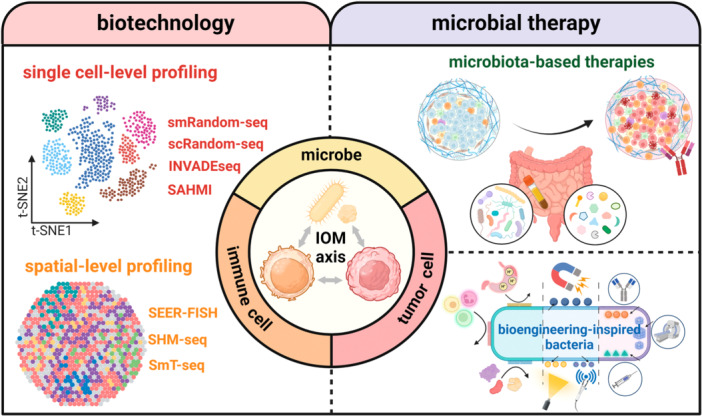

To the Editor,

Microbiota exists in symbiosis with the host, maintaining homeostasis and modulating immune function, whereas microbiota dysbiosis disrupts bodily functions, leading to diseases including cancers. With the evolution of biotechnology including sequencing methods, imaging techniques and culture‐based methods, numerous studies have provided insights into the mechanisms by which microbiota affect tumor progression, metastasis and therapeutic efficacy [1]. Aside from gut microbiota, recent investigations have focused on intratumor microbiota, which is recognized as an integral component of the tumor microenvironment (TME). The identification of this new component has introduced the next step of decoding TME towards the immuno‐oncology‐microbiome (IOM) axis, defined as the microbe‐mediated interactions among immune cells, tumor cells, and microbes within the TME [1].

16S rRNA sequencing and shotgun metagenomic sequencing, as mainstream methods to decipher the taxonomic composition and functional attributes of microbiome, have inherent technical limitations, such as low detection rates for rare species, ambiguous functional annotations, and absence of casual evidence [2], which can be compensated by multiple‐modality integrated methodologies. The emerging single‐cell and spatial profiling methods can reveal strain‐level genomic variations with high resolution and demonstrate the dynamic network interactions among microbiota, cancer cells and immunological components. Encouraging explorations in the IOM axis have sparked intense interest for utilizing microbiota‐based therapies to potentiate immunotherapy. Moreover, synthetic biotechnology and microbial engineering are also posing more possibilities for targeted and personalized regulations in cancer therapeutics.

## DECIPHERING THE IOM AXIS: FROM BULK TO SINGLE‐CELL AND SPATIAL LEVEL

**Figure 1:**
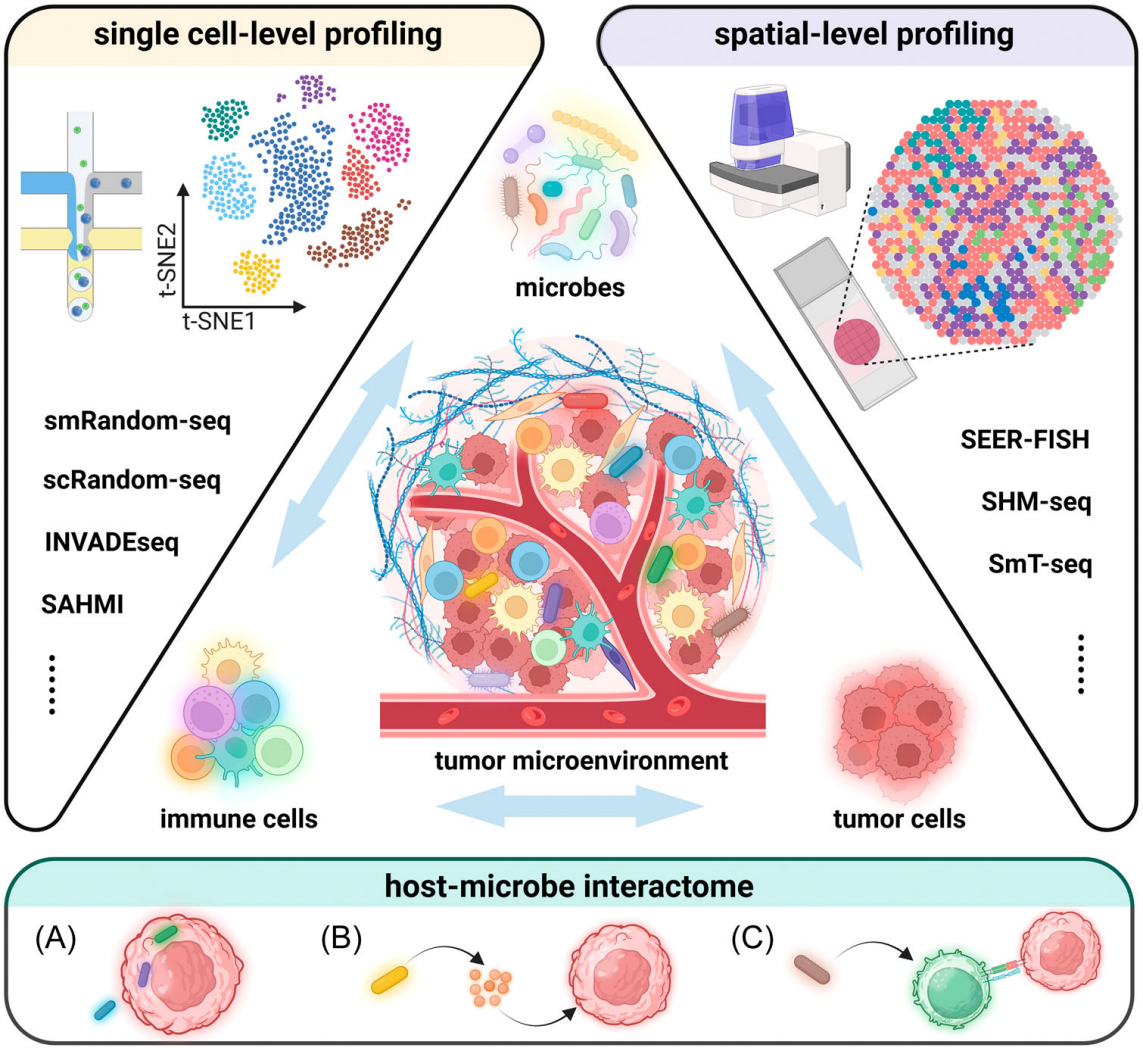
Single‐cell and spatial profiling approaches to decipher the multifaceted host‐microbe interactome. The advent of single‐cell and spatial profiling approaches allows for the precise analysis of individual host and microbial cells. In the context of single cell and spatial‐level profiling, we are able to decipher multi‐modal host‐microbe interactomes that has been overlooked in metagenomic sequencing. For example, (A) microbes may attach to and invade tumor cells to affect their immune phenotypes; (B) microbes can produce multiple substances to modulate TME distantly, including metabolites, extracellular vesicles, and so on; (C) microbes may act as the third party to break the balance between immune cells and tumor cells by regulating the differentiation and activation of immune cells. (Abbreviations: INVADEseq, invasion‐adhesion‐directed expression sequencing; SAHMI, single‐cell analysis of host‐microbiome interactions; SHM‐seq, spatial host‐microbiome sequencing; SmT‐seq, spatial metatranscriptomics sequencing).

Mounting studies have utilized multi‐omics approaches to depict the complicated microbe‐microbe or host‐microbe interactions. The underlying carcinogenic mechanisms can be classified into three types: intracellular microbes, microbial‐derived secretomes (e.g., metabolites, extracellular vesicles), and immunological modulation (Figure 1). The 16S rRNA gene and shotgun metagenomic sequencing technologies often overlook the single cell‐level functional heterogeneity and fail to capture spatial information. To overcome these limitations, a computational pipeline, termed as single‐cell analysis of host‐microbiome interactions (SAHMI), was proposed to discriminate microbial data from human‐derived single‐cell RNA sequencing (scRNA‐seq) reads [3]. It provides an optimized analytic workflow for mining the neglected microbial signals from the existing single‐cell sequencing databases. Additionally, diverse high‐throughput single‐cell sequencing methods with strain‐level resolution have been proposed (Figure 1). The smRandom‐seq exemplifies a solution by adding poly(dA) tails to the 3′hydroxyl terminus of in situ complementary DNAs (cDNAs) by virtue of terminal transferase (TdT) and innovatively utilizing a CRISPR‐based rRNA depletion method to enrich bacterial mRNA [4]. Compared with a previously published bacterial scRNA‐seq protocol named microSPLiT, smRandom‐seq achieved a significantly higher rate of mRNA capture efficiency (63% vs. 3.7%) and lower rRNA proportion (32% vs. 95.9%) [4]. Additionally, a high‐throughput cross‐species dual scRNA‐seq technology, termed as scRandom‐seq, captures both eukaryotic and bacterial RNAs and provides a research template for analyzing the dynamic changes in host cells at different infection stages [5]. This technique exhibits a highly efficient capacity for rRNA depletion (from 73.5% to 22.4%) and mRNA enrichment (from 12% to 44%) in both eukaryotic and prokaryotic samples [5].

Spatial microbiome profiling complementarily provides spatially resolved information about microbial distribution and interaction within tumors (Figure 1). Invasion‐adhesion‐directed expression sequencing (INVADEseq) is a modified scRNA‐seq method incorporating primers targeting 16S rRNA [6]. It reveals the heterogeneity of intratumor distribution of microorganisms. However, it merely partially demonstrates the nonhomogeneous distribution traits of intratumor microbiota without deeper mechanistic investigations. Recently, spatial host‐microbiome sequencing (SHM‐seq) was designed to spatially capture host and microbiome information simultaneously [7]. It combines spatial RNA and 16S rDNA sequencing to showcase the microbial biogeography and reveal the association of specific taxa with intestinal anatomical regions and host expression programs [7]. Aside from a high specificity of 97.0%, it sensitively captured all expected bacterial species in accord with the results of bulk RT‐qPCR [7]. As an example that successfully combines spatial sequencing and high‐resolution imaging, HiPR‐FISH, defined as high‐phylogenetic‐resolution microbiome mapping by fluorescence in situ hybridization, has revealed the intestinal spatial networks with the capacity of 1000‐fold multiplexity in taxa identification after a single round of imaging in about 5 min [8].

## MICROBIOTA‐BASED THERAPY: AN UNEXPECTED HELPER FOR IMMUNOTHERAPY

Immune checkpoint inhibitors (ICIs) have emerged as key players in reversing immunosuppressive TME. Notwithstanding, the interindividual heterogeneity in therapeutic response among patients restricts the favorable outcomes in a limited population. A deeper look into the underlying causes reveals that microbiota is an unanticipated yet non‐negligible element in remodeling local and distant tumor immune responses [1]. As such, exploring therapeutic modalities by rebuilding intestinal and intratumor microbiota presents a promising avenue to enhance immunotherapy effectiveness and extend its benefits to broader populations. Fecal microbiota transplantation (FMT) has shown promise in strengthening the suppressed immune system by restoring microbial homeostasis. Initial explorations of the immuno‐enhancing effectiveness of FMT primarily focused on melanoma. A multi‐center phase I trial demonstrated an overall response rate (ORR) of 65% when FMT was combined with pembrolizumab or nivolumab in patients with advanced‐stage cutaneous melanoma [9]. More recently, research has expanded beyond melanoma to explore its re‐sensitization effects on immunotherapy in gastrointestinal cancers, including gastric cancer, esophageal squamous cell carcinoma, and hepatocellular carcinoma. A study revealed that FMT achieved an ORR of 7.7% (1/13) and a disease control rate of 46.2% (6/13) in these cancers [10].

**Figure 2:**
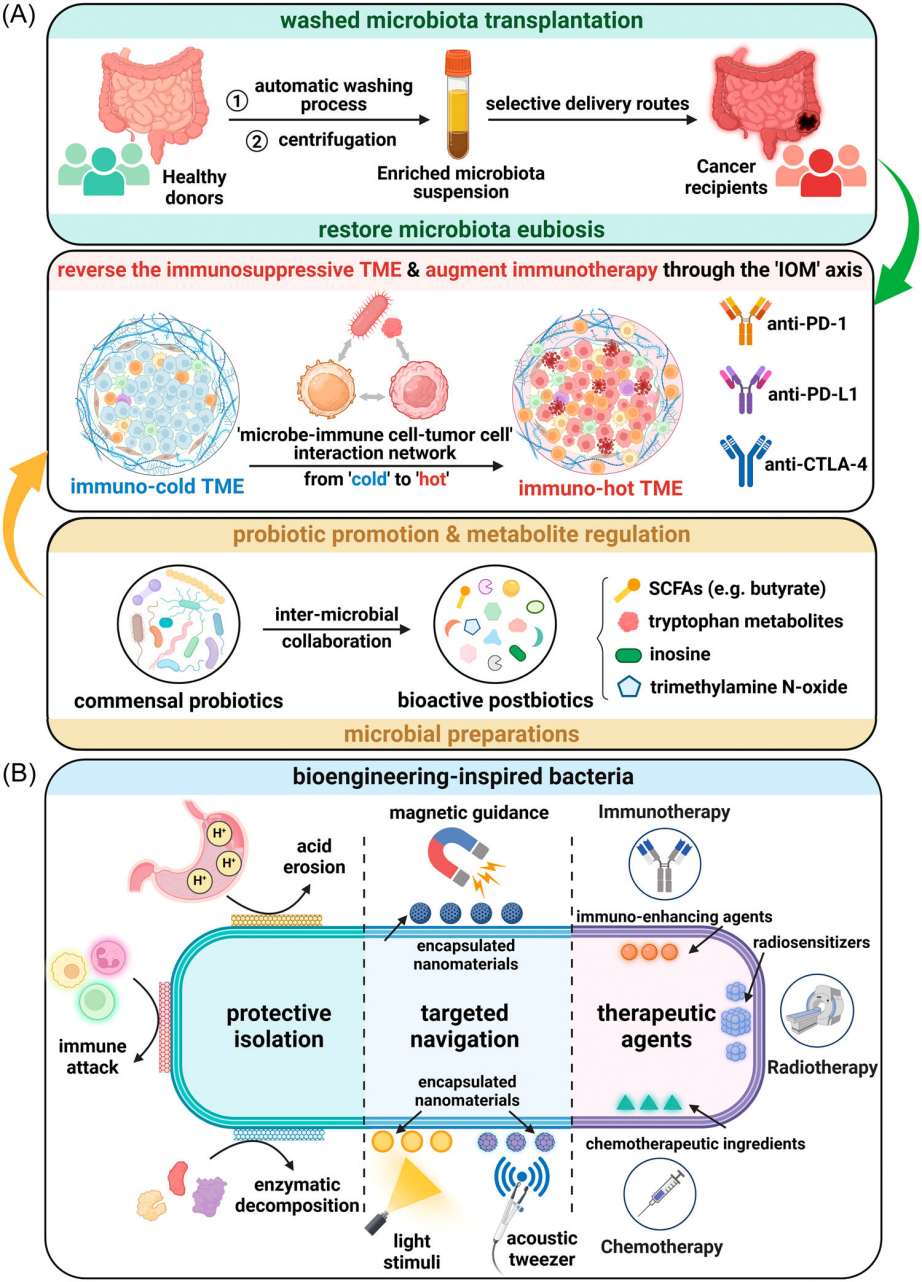
Microbially‐driven strategies for immunotherapy and targeted oncotherapy. (A) Washed microbiota transplantation has provided a quality‐controlled example of optimizing manual fecal microbiota transplantation. Delivery routes should be determined selectively to cope with the complex conditions of the gastrointestinal tract in cancer patients. Restoration of microbiota eubiosis contributes to balance the dysregulated IOM axis. Another microbiota‐based therapy is microbial preparations comprised of probiotics and postbiotics. Their supplementation may also modulate the “microbe‐immune cell‐tumor cell” interaction network to augment immunotherapy by reversing the TME from “cold” to “hot.” (B) Multiple modification approaches are applied to enable the engineered bacteria to reach the tumor stably and maximize their therapeutic effects. These approaches can be divided into three aspects comprised of protective isolation, targeted navigation and therapeutic agents. Camouflagic and protective encapsulations prevent bacteria from immunological and biochemical damage in vivo. For targeted navigation, nanoparticles with the capability of sensing magnetic or acoustic signals can be conjugated to the bacterial surface to ensure controllable translocation. The photoreceptor units can be utilized as light‐sensing switches to control the bacterial lysis for the release of loaded therapeutic agents, including immune‐enhancing agents, radiosentizers, chemotherapeutic ingredients, and so on. (Abbreviations: TME, tumor microenvironment; IOM axis, immuno‐oncology‐microbiome axis; anti‐PD‐1, anti‐programmed death‐1; anti‐PD‐L1, anti‐programmed death ligand‐1; anti‐CTLA‐4, anti‐cytotoxic T lymphocyte‐associated protein‐4; SCFAs, short‐chain fatty acids).

Nevertheless, the methodology of FMT provided by healthcare institutions varies in terms of donor‐sourced fecal processing, dosage and delivery routes. Methodological heterogeneity has hampered reproducibility and therapeutic quality controls. Current trends in the realm of FMT research can be categorized into technological innovation and procedural standardization. Washed microbiota transplantation (WMT) stands as the new method of manual FMT based on the automatic washing process and was released by the consensus statement from the FMT‐standardization Study Group in 2019 (Figure 2A) [11, 12]. Washed preparation of fecal microbiota improves the transplantation‐related safety, quantitative method, and delivery of microbiota suspension [11, 13]. Beyond a laboratory fecal disposal technique, FMT is a multi‐step medical service including donor selection, recipient evaluation and administration‐related factors (dosage, route, frequency, etc.). The establishment of uniform standards throughout the process will minimize inter‐study heterogeneity and facilitate longitudinal multi‐center clinical validation. Moreover, studies have highlighted its efficacy in ICI‐induced enterocolitis in cancer patients [14]. This evidence indicates that FMT holds the potential to “kill two birds with one stone” by treating gastrointestinal dysfunctions and potentiating immunotherapy simultaneously.

Another research trend in microbiota‐based therapies is to screen for probiotics and postbiotics to combat immunotherapy resistance (Figure 2A). A recent study demonstrated an example of inter‐microbial collaboration between *Lactobacillus johnsonii* and *Clostridium sporogenes* to produce indole‐3‐propionic acid (IPA), a tryptophan‐derived metabolite that activates CD8+ T cell‐mediated αPD‐1 immunotherapy [15]. In this study, oral administration of *Lactobacillus johnsonii* or IPA exerts similar immuno‐enhancing effects, highlighting the potential roles of probiotics and postbiotics in improving immunotherapy. Additionally, the diversity of microbial‐derived immuno‐potentiators suggests the potential for synergizing multiple probiotics and/or postbiotics in mixed formulations to maximize the immune‐enhancing efficacy, which points out the future research direction of synthetic microbiome consortia as a sustainable means of microbiota restoration post‐FMT. However, the actual efficacy and potential adverse effects of microbiota‐based therapy remain controversial and need to be validated on a larger preclinical scale. In combination with synthetic biotechnology, postbiotics can also be designed to be loaded on the engineered probiotics for targeted intratumor delivery and immune activation.

## ENGINEERING MICROBES FOR TARGETED CANCER THERAPY: TOWARDS PRECISION ONCOLOGY

Contemporary therapeutic protocols for cancer patients are plagued by their lack of tumor targeting and colonization capabilities, alongside with unfavorable efficacy, adverse events and increased healthcare costs. Besides immunotherapy, bioengineering technologies are considered to establish a robust foundation for targeted therapy and precision oncology. Microbes are ideal vectors for engineering due to their natural tropism and immunogenicity. In conjunction with advances in gene editing technology, the technical basis has been well established for modifying microbial genomes to selectively control biological actions, achieving a “1 + 1 > 2” effect.

Bacteria, the most commonly engineered microorganisms, are characterized by a fully editable structure. Modification strategies for engineered bacteria can be categorized into three aspects including protective isolation, targeted navigation and therapeutic agents (Figure 2B). Among these strategies, the priority is to protect microbes from the immunological and biochemical attacks in vivo. Multiple camouflagic and protective coatings such as cell membranes have achieved favorable isolation properties. For instance, “bacteria ghosts,” which are defined as empty shells of lysed bacteria without proliferation capability, hold great potential yet limited applicability in the field of bacterial cancer therapy for safety concerns brought by their retained immunogenicity, but fortunately, liposomal paclitaxel‐loaded bacterial ghosts encapsulated by cancer cell membranes have displayed higher tumor‐targeting properties and antitumor toxicity together with ensured safety [16]. The purpose of next decoration is to achieve noninvasive and targeted manipulation of in vivo movement and intratumor lysis. Based on the navigability of magnetotactic bacteria, magnetic nanomaterials can be bioengineered onto bacterial surfaces to artificially control tumor‐targeted movement under directional magnetic fields [17]. Acoustic tweezers are also applied to enable programmable movement in the circulatory system by virtue of tissue‐penetration capacity of ultrasound [18]. To control bacterial lysis and subsequent drug release, the engineered light‐sensing units are often utilized as switches. For example, ICI‐loaded bacteria can produce an intratumor immune activation effect with the combination of photothermal stimulation [19]. Similarly, chemotherapeutic agents or radiation sensitizers can also be loaded on microbes to enhance local therapeutic effects and reduce systemic toxicity [19]. Exploring multi‐modal integrative therapies for cancer treatment will provide a methodological framework for addressing the unsatisfactory efficacy of monotherapy.

Looking ahead, challenges facing the widespread application of engineered microbial therapies extend beyond scientific innovation. Considerations for safety and cost‐effectiveness are indispensable. The actual efficacy and safety properties of engineered microbes have rarely been further replicated. Analogous to introducing exotic species in the macro world, engineered microbial therapies highlight the consideration of potential microecological perturbations resulting from gene‐edited microbes. High expense of engineering design remains another translational obstacle. The example of chimeric antigen receptor (CAR)‐T cell therapy demonstrates the importance of addressing cost barriers to ensure widespread accessibility of these cutting‐edge therapies.

## CONCLUSIONS AND PROSPECTS

As microbiome sequencing technology advances, recent studies have extended the boundaries of species to subspecies by investigating strain‐level genetic mutations and their impacts on tumors. For instance, a distinct clade within *Fn* subspecies, termed as *Fn* subspecies *animalis* (*Fna*) C2, was identified to predominantly enrich in colorectal cancer niches [20], indicating that different subspecies, featuring similar “core genome” and distinct “accessory genome,” have disparate effects on tumorigenesis.

Challenges ahead of microbiota‐based therapies include issues of safety, acceptance and reproducibility. These concerns prompt several critical questions: (i) What strategies can be implemented to mitigate short‐term safety risks associated with microbiota‐based therapies? (ii) What are the potential long‐term consequences of microbiota modulation, such as chronic disease transmission? (iii) How can the reproducibility of microbiota‐based interventions be enhanced to ensure consistent outcomes across studies? (iv) In what ways do specific gut microbial taxa modulate ICI efficacy across cancer types? Is it drug‐specific or tumor‐specific? (v) How can microbiota‐based therapies be effectively integrated into clinical practice and harmonized with existing therapies? Considering these issues, longitudinal prospective safety cohorts are essential for evaluating the sustained impact of these therapies on patient health.

Moving forward, we should be wary of the translational bottlenecks arising from poorly regulated technical commercialization. Microbiota‐based therapies are often classified as biological products; however, they are categorized differently across regions. In the United States and Canada, they are considered investigational drugs, whereas in Italy, the Netherlands, and Belgium, they are defined as tissue transplants. These classification differences underscore more comprehensive investigations of the IOM axis. Accordingly, collaboration among researchers, clinicians, regulators, and industry stakeholders is essential to advance microbiome profiling technologies and microbiota‐based therapies from bench to bedside, potentially revolutionizing tumor diagnosis and treatment in the upcoming decades.

## AUTHOR CONTRIBUTIONS

**Zheshun Pi**: Writing—original draft; Writing—review and editing. **Weici Liu**: Writing—original draft; Writing—review and editing. **Chenghu Song**: Investigation. **Chuandong Zhu**: Investigation. **Jiwei Liu**: Investigation. **Lu Wang**: Investigation. **Zhao He**: Investigation. **Chengliang Yang**: Investigation. **Lei Wu**: Investigation. **Tianshuo Liu**: Investigation. **Zijie Geng**: Investigation. **Scott J. Tebbutt**: Conceptualization; Writing—review and editing; Supervision. **Ningning Liu**: Conceptualization; Writing—review and editing; Supervision. **Yuan Wan**: Conceptualization; Writing—review and editing; Supervision; Funding acquisition. **Faming Zhang**: Conceptualization; Writing—review and editing; Supervision. **Wenjun Mao**: Writing—review and editing; Conceptualization; Supervision; Funding acquisition.

## CONFLICT OF INTEREST STATEMENT

Faming Zhang conceived the concept of GenFMTer and transendoscopic enteral tubing and the devices (FMT Medical, Nanjing, China) related to them. The remaining authors declare no conflict of interest.

## ETHICS STATEMENT

No animals or humans were involved in this study.

## Data Availability

Data sharing not applicable to this article as no datasets were generated or analyzed during the current study. No new data were generated in this study. Supplementary materials (graphical abstract, slides, videos, Chinese translated version, and update materials) may be found in the online DOI or iMeta Science http://www.imeta.science/.
